# Upregulation of PGC-1*α* Attenuates Oxygen-Glucose Deprivation-Induced Hippocampal Neuronal Injury

**DOI:** 10.1155/2022/9682999

**Published:** 2022-06-09

**Authors:** Bin Han, Hui Zhao, Xingji Gong, Jinping Sun, Song Chi, Tao Liu, Anmu Xie

**Affiliations:** ^1^Department of Neurology, The Affiliated Hospital of Qingdao University, Qingdao 266000, China; ^2^Department of Cardiology, The Affiliated Hospital of Qingdao University, Qingdao 266000, China; ^3^Department of Emergency Internal Medicine, The Affiliated Hospital of Qingdao University, Qingdao 266000, China

## Abstract

Hippocampal neuronal damage likely underlies cognitive impairment in vascular dementia (VaD). PPAR*γ* coactivator-1*α* (PGC-1*α*) is a master regulator of mitochondrial biogenesis. However, the role and the precise mechanism of how PGC-1*α* alleviates hippocampal neuronal injury remain unknown. To address this question, HT-22 cells, an immortalized hippocampal neuron cell line, with or without PGC-1*α* overexpression were subjected to oxygen-glucose deprivation (OGD), which mimics the circumstance of chronic cerebral hypoperfusion in VaD. After OGD, cell viability was assessed using the MTS assay. The mitochondrial function and reactive oxygen species (ROS) were both detected. ChIP-Seq analysis was employed to discover the underlying molecular mechanism of PGC-1*α*-mediated neuroprotective effects. Our results showed that mitochondrial membrane potentials were increased and ROS production was decreased in PGC-1*α* overexpressing cells, which increased cell viability. The further bioinformatics analysis from ChIP-Seq data indicated that PGC-1*α* may participate in the regulation of apoptosis, autophagy, and mitophagy pathways in HT-22 cells. We found that PGC-1*α* promoted the LC3-II formation and reduced the neuronal apoptosis determined by TUNEL staining. In addition, PGC-1*α* upregulated the expressions of mitochondrial antioxidants, including SOD2, Trx2, and Prx3. In summary, our findings indicate that PGC-1*α* may attenuate OGD-induced hippocampal neuronal damage by regulating multiple mechanisms, like autophagy and mitochondrial function. Thus, PGC-1*α* may be a potential therapeutic target for hippocampal damage associated with cognitive impairment.

## 1. Introduction

Vascular dementia (VaD) is considered second only to Alzheimer's disease in the type of dementia [1]. In China, the prevalence of VaD is expected to increase as the population ages [2]. Many factors, such as oxidative stress, mitochondrial dysfunction, neuronal damage, and neuroinflammation, all contribute to the pathology of VaD [3]. Dementia is closely related to stroke, which is bringing an increasing burden of disease [4]. Since 2010, stroke accounts for the majority of deaths and disabilities in China [5]. The risk of stroke is increasing, and stroke interacts with risk factors for dementia. Reducing risk factors for stroke and dementia is a huge public health challenge that requires urgent action. Disappointingly, current strategies could not effectively prevent or slow the progression of dementia [3]. Therefore, it is urgent to continue to explore other effective treatments.

In the past several decades, the hippocampus has been tightly linked to cognitive functions, including memory and learning [6]. Indeed, substantial evidence has showed that hippocampal volume was reduced in VaD patients compared to normal control [7, 8]. For animal experiments, hippocampal atrophy could occur in a mouse model of bilateral common carotid artery stenosis using microcoils, which has cognitive impairment, and mimics the pathology of VaD quite well [9]. It is well known that damage to hippocampal neurons is commonly accompanied by oxidative stress and mitochondrial dysfunction under chronic cerebral hypoperfusion [10, 11]. The target to restore oxidative stress and mitochondrial dysfunction has been proved to alleviate cognitive impairments [12]. Therefore, finding ways to alleviate hippocampal neuron damage may be a potentially effective treatment option for improving cognitive function.

We previously found that upregulation of neuronal peroxisome proliferator-activated receptor-*γ* coactivator-1*α* (PGC-1*α*) generated from PGC-1*α*^f/f^ Eno2-Cre mice ameliorates cognitive impairment induced by chronic cerebral hypoperfusion [13]. However, the precise mechanism of how PGC-1*α* affects hippocampal neuronal responses is still unclear. Therefore, it should be meaningful to uncover the molecular function of PGC-1*α* in hippocampal neurons, thereby revealing better ways of regulation to improve cognitive function for VaD. PGC-1*α*, a transcriptional coactivator, has been viewed as a master regulator of mitochondrial biogenesis [14]. Mitochondrial dysfunction in neurons occurs in the early stage after ischemia. Thus, mitochondrial biogenesis-based therapy may be a powerful potential therapeutic target for neurological diseases [15, 16]. We assumed that PGC-1*α* improved mitochondrial function, thereby protecting hippocampal neurons against damage from chronic cerebral hypoperfusion.

In this study, we used HT-22 cells, an immortalized hippocampal neuron cell line, to detect the underlying mechanism of PGC-1*α* by combining bioinformatics analysis. Then, we further verified the molecular function of PGC-1*α* using multiple experimental methods.

## 2. Materials and Methods

### 2.1. Cell Culture and Lentiviral Transfection

HT-22 cells were cultured in Eagle's Minimum Essential Medium (EMEM) (10-009-CVR, Corning) containing 10% fetal bovine serum (FBS, Life Technologies, Vienna, Austria) and 1% penicillin/streptomycin (Life Technologies) in a sterile cell incubator at 37°C with 5% of CO_2_. To overexpress PGC-1*α* in HT-22 cells, we employed lentivirus transfection technology. Firstly, we amplified the target gene using the following primers: 5′-GAGGATCCCCGGGTACCGGTCGCCACCATGGCTTGGGACATGTGCAG−3′ (forward) and 5′-TCCTTGTAGTCCATACCCCTGCGCAAGCTTCTCTGAGCTTC−3′ (reverse). Then, we chose the lentiviral vector Ubi-MCS-3FLAG-SV40-EGFP-IRES-puromycin to clone the target gene. Similarly, a negative control was also cloned into the same lentiviral vector. After that, HT-22 cells were transfected with the constructed lentiviruses. The transfection effect could be monitored by a green fluorescent label with EGFP. The target gene or negative control was successfully integrated into the genome of HT-22 cells. The successfully transfected HT-22 cell would express the protein of EGFP. To further screen the transfected HT-22 cells, puromycin (2 *μ*g/ml; Santa Cruz) was added into the medium at 72 h after transfection. The cells that had failed to be transfected would be screened out. The screened HT-22 cells would be used for the subsequent experiments.

### 2.2. Western Blots

The transfected HT-22 cells were harvested. Then, total protein was extracted from HT-22 cells lysed with RIPA buffer (Solarbio, Beijing, China) containing cOmplete™ protease inhibitor cocktail (Roche). The protein concentration was measured using the BCA kit (Solarbio, Beijing, China) according to the manufacturer's guidelines. An equal amount of protein was loaded into SDS-PAGE gel. After separation, the protein was transferred to a PVDF membrane (Millipore, USA). The membrane was blocked with 5% nonfat dried milk for 2 h at room temperature and then incubated with primary antibodies, including rabbit anti-PGC-1*α* (1 : 1000, ThermoFisher) and rabbit anti-beta-actin (1 : 1000, Abcam), overnight at 4°C. In the next step, the membrane was incubated with the species-appropriate secondary antibodies for 1 h at room temperature. After washing, the bands were captured with Bio-Rad Gel Doc Imager and analyzed using ImageJ software.

### 2.3. Oxygen-Glucose Deprivation (OGD) Treatment

To mimic hypoxia injury, HT-22 cells were cultured in the Hypoxia Incubator Chamber (Cat. 27310; STEMCELL Technologies Inc), a self-contained and sealed chamber, as we described previously [13, 17]. Briefly, HT-22 cells, carrying the target gene and negative control, were cultured in DMEM without serum and glucose and were immediately transferred into the chamber (5% CO_2_ and 95% N_2_) for 2 h. Then, DMEM was replaced by complete medium. Cells were cultured in the sterile cell incubator at 37°C with 5% of CO_2_ for 24 h.

### 2.4. Cell Viability Assay

The 3-(4,5-dimethylthiazol-2-yl)-5-(3-carboxymethoxyphenyl)-2-(4-sulfophenyl)-2H-tetrazolium (MTS) assay was used to determine the viability of HT-22 cells according to the manufacturer's instructions. Briefly, HT-22 cells with or without PGC-1*α* overexpression were cultured in a 96-well assay plate at a density of 10^4^ cells per well. After OGD treatment, MTS/PMS solution (20 *μ*l) was added to each well containing 100 *μ*l of cells in culture medium. Then, the culture plate was placed in the sterile cell incubator at 37°C with 5% of CO_2_ for 2 h. Finally, the absorbance was recorded at 490 nm using the Thermo Scientific Microplate Reader.

### 2.5. Determination of Mitochondrial Function

To evaluate the mitochondrial function, Mito-Tracker Red CMXRos (Beyotime Institute of Biotechnology, Nanjing, China), a red-fluorescent dye, was employed to measure the mitochondrial membrane potential in live cells and thus reflect mitochondrial activity. After OGD treatment, HT-22 cells with or without PGC-1*α* overexpression were incubated with Mito-Tracker Red CMXRos working solution (100 nM) for 20 min at 37°C. The working solution was removed, and fresh cell culture medium preincubated at 37°C was added. Finally, the mean fluorescence intensity was quantified by a fluorospectrophotometer. The excitation wavelength is 579 nm, and the emission wavelength is 599 nm.

### 2.6. Detection of Reactive Oxygen Species (ROS) Production

The Reactive Oxygen Species Assay kit (Beyotime Institute of Biotechnology, Nanjing, China) was used to detect the level of ROS in HT-22 cells with or without PGC-1*α* overexpression according to the manufacturer's instructions. In brief, HT-22 cells were seeded in a 96-well plate and underwent OGD treatment. After removing the culture medium, HT-22 cells were incubated with a fluorimetric probe, 2′,7′-dichlorodihydrofluorescein-diacetate (DCFH-DA) (10 *μ*M), in the sterile cell incubator at 37°C with 5% of CO_2_ for 20 min. Subsequently, HT-22 cells were washed 3 times using serum-free medium. Finally, the mean fluorescence intensity was quantified by a fluorospectrophotometer. The excitation wavelength is 488 nm, and the emission wavelength is 525 nm.

### 2.7. Chromatin Immunoprecipitation-Sequencing (ChIP-Seq) Assay

To reveal the molecular mechanism of the neuroprotective effect of PGC-1*α* on HT-22 cells, ChIP-Seq was employed to identify the targets of PGC-1*α*. After OGD treatment, HT-22 cells were harvested and centrifuged. The supernatant was removed, and formaldehyde was added to HT-22 cells to cross-link proteins to DNA. Then, chromatin was extracted and sheared by sonication. PGC-1*α* antibody (NBP1-04676, NOVUS) was used for immunoprecipitation to acquire the protein of interest. The DNA samples were harvested and purified. After that, 10 ng DNA was converted to phosphorylated blunt-ended followed by an “A” base added to the 3′ end of the blunt phosphorylated DNA fragments. Illumina's genomic adapters were ligated to the fragments, which were further amplificated by PCR. The selected ~200-1500 bp product was used for sequencing. The generated single-stranded DNA was amplified using the HiSeq 3000/4000 PE Cluster Kit (PE-410-1001, Illumina). Next, the DNA was sequenced on Illumina HiSeq 4000 according to the manufacturer's instructions. After sequencing, the base calling was performed using Off-Line Basecaller software (OLB V1.8). The sequence quality was evaluated by the Solexa CHASTITY quality filter. Then, the clean reads were aligned to mouse reference genome UCSC MM10 using BOWTIE (V2.1.0). The peak calling was detected using MACS V1.4.2, and peak annotation was acquired using the newest UCSC RefSeq database. The peaks located within -2 kb to +2 kb around the corresponding gene TSS were selected and identified as promoter-centered annotation. These genes were further used for GO term analysis and KEGG pathway analysis.

### 2.8. Immunofluorescence Staining

HT-22 cells were planted on poly-L-lysine coated glass coverslips and cultured in the Hypoxia Incubator Chamber. After OGD treatment, HT-22 cells were permeabilized with 0.3% Triton X-100 for 10 min at room temperature and then washed with PBS for 3 times. In order to reduce nonspecific staining, HT-22 cells were blocked with 3% BSA for 1 h. After washing, the cells were incubated with the primary antibodies, including rabbit anti-LC3 (1 : 200, #4108, Cell Signaling Technology), rabbit anti-SOD2 (1 : 150, NB100-1992, NOVUS), rabbit anti-Trx2 (1 : 200, ab185544, Abcam), and rabbit anti-Prx3 (1 : 100, A3076, ABclonal), overnight at 4°C. On the second day, the primary antibodies were removed and washed 5 times with PBS. HT-22 cells were incubated with Alexa Fluor 488-conjugated donkey anti-rabbit secondary antibody (1 : 500; A-21206; Thermo Fisher Scientific) for 1 h at room temperature in the dark. After washing, the cell nucleus was stained with DAPI (ab104139, Abcam). Finally, the images were captured with a fluorescent microscope and analyzed with ImageJ software.

### 2.9. In Situ Cell Death Detection

To evaluate the effect of PGC-1*α* on the apoptosis of HT-22 cells, terminal transferase-mediated dUTP nick end labeling (TUNEL) staining was used to perform in situ cell death detection. HT-22 cells were processed as described in the above protocol. After permeabilization and blocking, HT-22 cells on the coverslips were incubated with TUNEL reaction mixture (5 *μ*l enzyme solution and 45 *μ*l label solution per sample) at 37°C for 1 h in the dark from the In Situ Cell Death Detection Kit (Roche) according to the manufacturer's guidelines. After washing and DAPI staining, the samples were analyzed by a fluorescent microscope. The percentage of apoptosis was calculated.

### 2.10. Statistical Analysis

All data were analyzed using GraphPad Prism (GraphPad Software, Version 5.0, La Jolla, CA, USA) and shown as means ± SEM. The difference between two groups was compared with the Mann–Whitney *U* test. *p* < 0.05 was considered statistically significant.

## 3. Results

### 3.1. PGC-1*α* Overexpression Protects Hippocampal Cells against OGD-Induced Damage

**Figure 1:**
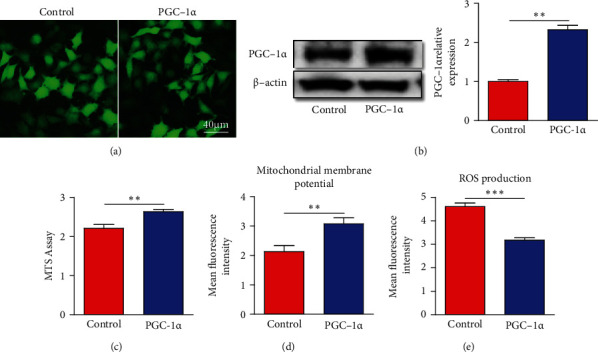
PGC-1*α* protect against OGD-induced hippocampal neuron damage. (a) Representative images of EGFP expression in HT-22 cells after lentivirus transfection. (b) Western blot showing PGC-1*α* expression in control group and PGC-1*α* group (left panel). The relative expression of PGC-1*α* was measured using ImageJ software (right panel). (c) MTS analysis showing the effect of PGC-1*α* on viability of HT-22 cells. (d) The evaluation of PGC-1*α* on mitochondrial function reflected by mitochondrial membrane potential. (e) Quantitation of ROS production in HT-22 cells after OGD treatment. ^∗∗^*p* < 0.01,  ^∗∗∗^*p* < 0.001; *n* = 5-10 per group.

To assess the effect of PGC-1*α* on hippocampal cells upon hypoxia injury, PGC-1*α* lentivirus was constructed and transfected into HT-22 cells. As shown in Figure 1(a), the stable HT-22 cell line was established after purinomycin screening, and all HT-22 cells expressed the protein of EGFP. To evaluate the efficiency of PGC-1*α* overexpression, western blot was employed, and we found that the expression of PGC-1*α* in the PGC-1*α* group was approximately twofold higher than that in the control group (Figure 1(b)). To further investigate the protective effect of PGC-1*α* on HT-22 cells, we found that PGC-1*α* overexpression increased the cell viability using MTS analysis after OGD treatment (Figure 1(c)). In addition, we found that PGC-1*α* improved the mitochondrial membrane potential of HT-22 cells under the hypoxia environment (Figure 1(d)). Further analysis indicated that PGC-1*α* overexpression reduced the production of ROS (Figure 1(e)). Taken together, these findings suggest that PGC-1*α* may improve mitochondrial function and reduce ROS levels, thereby alleviating chronic cerebral hypoperfusion-induced hippocampal cell injury.

### 3.2. Identification of Genome-Wide Transcriptional Targets of PGC-1*α* in HT-22 Cells after OGD Treatment

**Figure 2:**
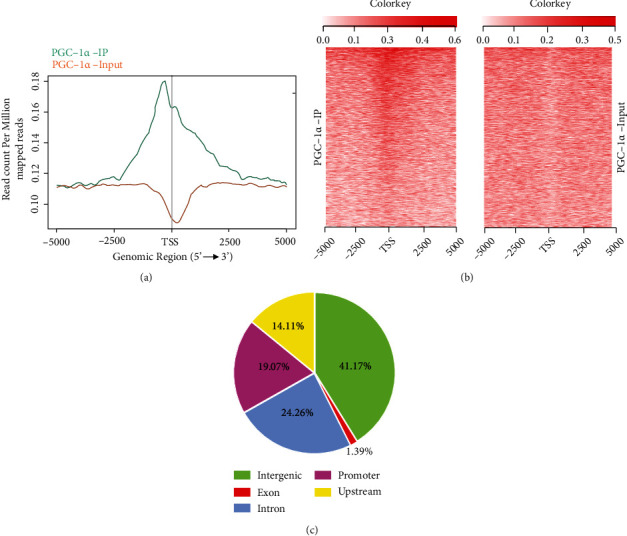
Identification of genome-wide transcriptional targets of PGC-1*α* in HT-22 cells. (a) The density distribution for ChIP-Seq peaks of PGC-1*α*. (b) Heat maps in PGC-1*α*-IP vs. PGC-1*α*-input; (c) ChIP-Seq peaks of PGC-1*α* distributed over important genomic features.

To reveal the precise molecular mechanism of PGC-1*α* and how to regulate HT-22 cell functions, we used ChIP-Seq analysis to identify the potential targets of PGC-1*α* in HT-22 cells under hypoxia circumstances. After sequencing, the reads were aligned to the mouse genome, and the mapped reads were used for peak detection. The distribution of statistically significant ChIP-enriched peaks, annotated by the nearest gene around TSS (±5 kb to TSS), was plotted as shown in Figure 2(a). In addition, the heatmaps of peak distribution were both displayed (Figure 2(b)). Based on the distances of peaks to UCSC RefSeq genes, the peaks were divided into five classes, including 47.17% in intergenic regions, 24.26% in intron regions, 14.11% in upstream regions, 1.39% in exon regions, and 19.07% in promoter regions, which were located within -2 kb to +2 kb around the corresponding gene TSS (Figure 2(c)).

### 3.3. Bioinformatics Analysis Reveals That PGC-1*α* May Play a Neuroprotective Role in Many Ways

**Figure 3:**
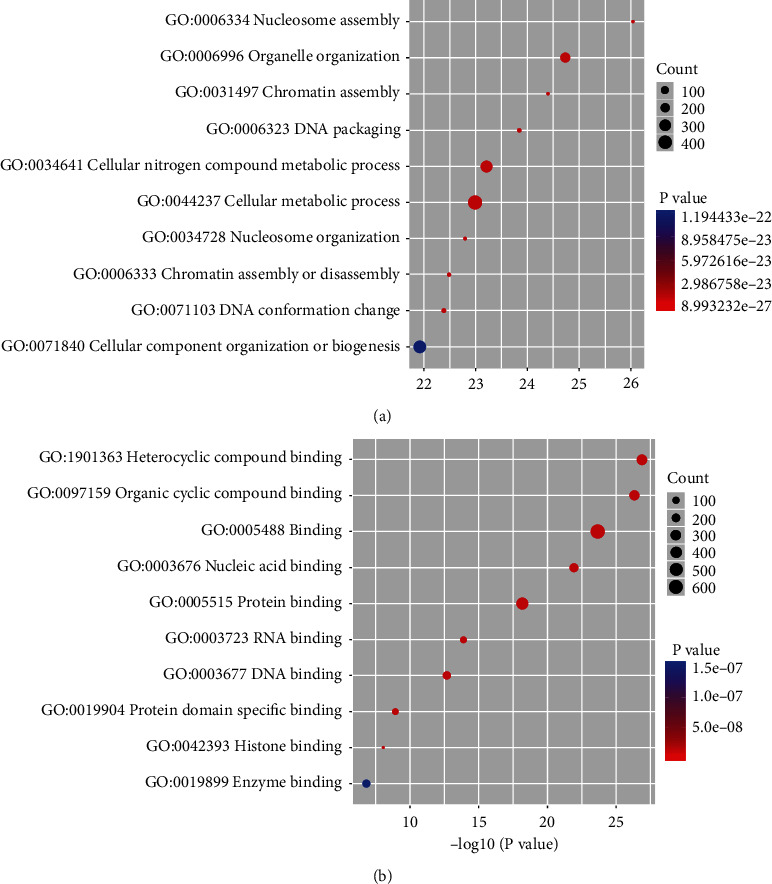
Gene Ontology analysis based on promoter-centered annotation. GO enrichment analysis on peaks which were located within -2 kb to +2 kb around the corresponding gene TSS. (a) The top ten enriched biological processes were shown. (b) The top ten enriched molecular functions were shown.

Considering that PGC-1*α* plays a regulatory role in combination with promoter regions of genes, we, thus, further analyzed the peaks distributed in promoter regions. GO analysis revealed the top ten of biological process, including nucleosome assembly, organelle organization, chromatin assembly, DNA packaging, cellular nitrogen compound metabolic process, cellular metabolic process, nucleosome organization, chromatin assembly or disassembly, DNA conformation change, and cellular component organization or biogenesis (Figure 3(a)). These biological processes were all closely related to cellular metabolism. In addition, the top ten of molecular function were also shown, including heterocyclic compound binding, organic cyclic compound binding, binding, nucleic acid binding, protein binding, RNA binding, DNA binding, protein domain specific binding, histone binding, and enzyme binding (Figure 3(b)), which were all consistent with the physiological function of PGC-1*α* as a master coregulator.

**Figure 4:**
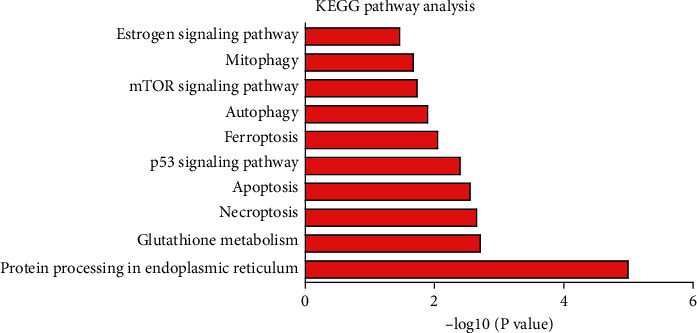
Kyoto Encyclopedia of Genes and Genomes (KEGG) pathway analysis discovers the biological pathway. The top ten enriched pathways were shown.

To further reveal the signaling pathway of participation, KEGG pathway analysis was employed, and the highly enriched signaling pathways were protein processing in endoplasmic reticulum, glutathione metabolism, necroptosis, apoptosis, p53 signaling pathway, ferroptosis, autophagy, mTOR signaling pathway, mitophagy, and estrogen signaling pathway (Figure 4). These findings suggest that PGC-1*α* may play the neuroprotective role of hippocampal neurons through multiple mechanisms.

### 3.4. PGC-1*α* Promotes Autophagy and Reduces Apoptosis in Hippocampal Cells

**Figure 5:**
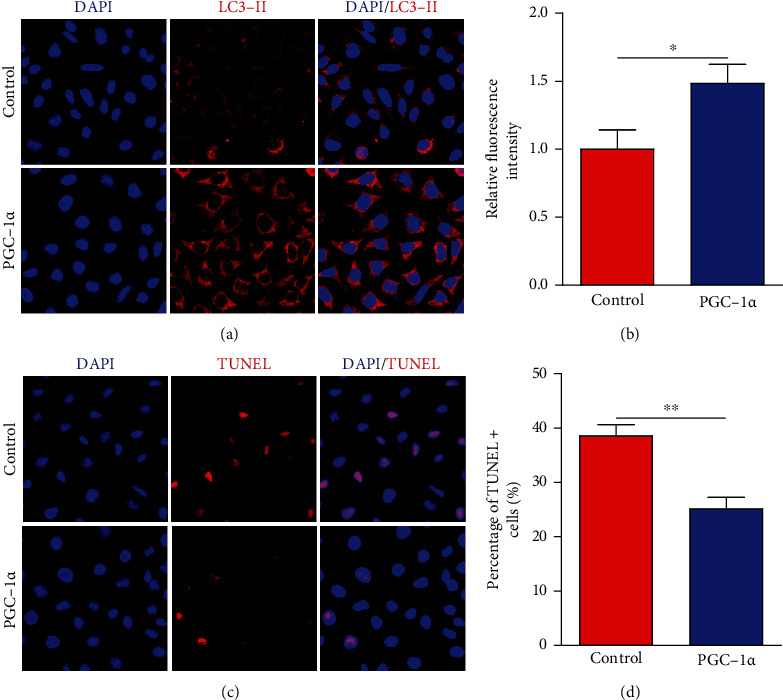
PGC-1*α* promotes autophagy and reduces hippocampal neuron apoptosis. (a) Representative images of LC3-II level in HT-22 cells with or without PGC-1*α* overexpression after OGD treatment. (b) Quantification of the relative fluorescence intensity of LC3-II using ImageJ software. (c) Analysis of hippocampal neuron apoptosis using TUNEL staining after OGD treatment. (d) Quantification of the percentage of TUNEL^+^ cells. ^∗^*p* < 0.05,  ^∗∗^*p* < 0.01; *n* = 8 per group.

To further verify the results derived from the ChIP-Seq data, we evaluated the effect of PGC-1*α* on autophagy and apoptosis in HT-22 cells. Many studies have demonstrated that autophagy, clearance of damaged organelles and misfolded or aggregated proteins, plays crucial roles in neurons, like participating in the neuronal development, homeostasis, plasticity, and neurotransmission [18–20]. For ChIP-Seq analysis in our study, the pathway of autophagy was also highly enriched. Indeed, we found that the expression of LC3-II was increased in HT-22 cells with PGC-1*α* overexpression (Figure 5(a)). The relative fluorescence intensity of LC3-II was also higher in the PGC-1*α* group compared with that in the control group (Figure 5(b)). Furthermore, the impact of PGC-1*α* on apoptosis was assessed. We found that PGC-1*α* effectively reduced the apoptosis of HT-22 cells (Figures 5(c) and 5(d)). Altogether, these results suggest that PGC-1*α* promotes autophagy, which may potentially contribute to reducing apoptosis and hippocampal neuron damage.

### 3.5. PGC-1*α* Reduces Oxidative Stress by Modulating Mitochondrial Antioxidants in Hippocampal Cells

**Figure 6:**
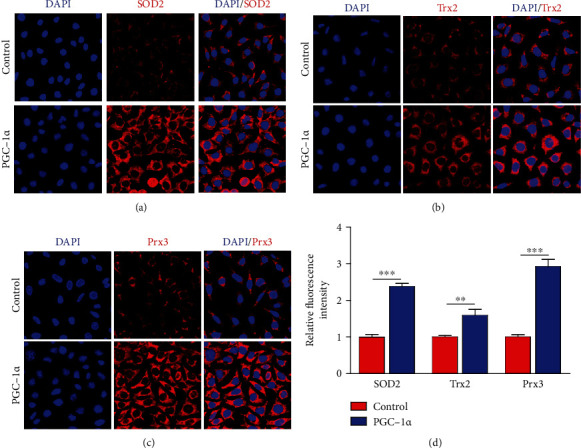
PGC-1*α* promotes the expression of mitochondrial antioxidants in hippocampal cells. (a) Representative images of SOD2 expression in HT-22 cells with or without PGC-1*α* overexpression after OGD treatment. (b) Representative images of Trx2 expression in HT-22 cells with or without PGC-1*α* overexpression after OGD treatment. (c) Representative images of Prx3 expression in HT-22 cells with or without PGC-1*α* overexpression after OGD treatment. (d) Quantification of the relative fluorescence intensity of SOD2, Trx2, and Prx3 using ImageJ software. ^∗∗^*p* < 0.01,  ^∗∗∗^*p* < 0.001; *n* = 8 per group.

It has been well known that PGC-1*α* plays important roles in the mitochondrial antioxidant defense system, such as superoxide dismutase 2 (SOD2), thioredoxin 2 (Trx2), and peroxiredoxin 3 (Prx3). SOD2 reduces ROS production by converting the superoxide byproducts of oxidative phosphorylation. In addition, Trx2 and Prx3 suppress mitochondrial ROS generation by regulating cellular redox and playing the scavenging role. Based on this, we also evaluated whether PGC-1*α* participates in the regulation of mitochondrial antioxidants in hippocampal cells. Immunofluorescence analysis indicated that PGC-1*α* promoted the expression of SOD2 (Figure 6(a)). The relative fluorescence intensity showed that PGC-1*α* increased the expression of SOD2 by about 2.3 times (Figure 6(d)). We also found that PGC-1*α* approximately increased the expression of Trx2 by 1.6 times (Figures 6(b) and 6(d)). Moreover, the expression of Prx3 increased about 2.9 times in HT-22 cells from the PGC-1*α* group than that from the control group (Figures 6(c) and 6(d)). These data strongly indicate that regulating oxidative stress also contributes to the neuroprotective effect of PGC-1*α* on hippocampal neurons under hypoxia environment.

## 4. Discussion

In this study, we detected the underlying mechanism of PGC-1*α* in hippocampal neurons using HT-22 cells. Under hypoxia environment, PGC-1*α* overexpression effectively enhanced mitochondrial function and reduced ROS production, thereby improving the survival rate of HT-22 cells. Based on ChIP-Seq analysis, the potential targets of PGC-1*α* in HT-22 cells were identified. KEGG pathway analysis indicated that PGC-1*α* may play neuroprotective roles through various mechanisms, such as regulating apoptosis, autophagy, and ferroptosis. Indeed, we observed that PGC-1*α* promoted the expression of autophagy-related protein and mitochondrial antioxidants and reduced cell apoptosis. Thus, our findings highlight that multiple potential mechanisms may confer neuroprotection of PGC-1*α* in hippocampal neurons.

PGC-1*α* has been identified as a master regulator of mitochondrial biogenesis and function [21] and has also been associated with many important functions, such as modulating cellular energy, oxidative stress, and inflammation [15, 16, 22]. In the cortex of patients with multiple sclerosis, the level of neuronal loss was correlated with reduced PGC-1*α* expression, which was accompanied by lower mitochondrial function and increased ROS production [23]. In addition, PGC-1*α* overexpression in astrocytes markedly suppressed the level of proinflammatory cytokines, including IL-6 and CCL2 [24]. In microglia, PGC-1*α* also participated in the anti-inflammatory reaction, such as inhibition of NF-kappaB activity, regulation of microglial polarization, promotion of autophagy and mitophagy, and suppression of NLRP3 inflammasome-induced inflammatory responses [17, 25, 26]. Moreover, a previous study indicated that exercise could promote the BDNF expression of hippocampal neurons through PGC-1*α*, which, in turn, improved cognitive function [27]. Thus, it may have important implications for revealing the precise mechanism of PGC-1*α* in chronic cerebral hypoperfusion-induced hippocampal neuron damage.

In our study, we used ChIP-Seq analysis to identify the potential targets of PGC-1*α* in HT-22 cells after OGD treatment. Based on ChIP-Seq data, KEGG pathway analysis discovered several highly enriched pathways, including protein processing in the endoplasmic reticulum, glutathione metabolism, necroptosis, apoptosis, p53 signaling pathway, ferroptosis, autophagy, mTOR signaling pathway, mitophagy, and estrogen signaling pathway. Several researches have demonstrated that modulation of protein processing in the endoplasmic reticulum was closely related to the apoptosis of hippocampal neurons [28–30]. Glutathione metabolism and ferroptosis were both thought to be related to hippocampal neuron death [31–34]. Gao et al. indicated that activation of the p53 signaling pathway could ameliorate hippocampal neuron damage, thereby improving learning and memory of hypoxia/reoxygenation-induced brain injury in rats [35]. In addition, the mTOR signaling pathway was considered to participate in the process of autophagy. The two physiological processes both contribute to the protection of hippocampal neurons [36–38]. We showed that PGC-1*α* improved mitochondrial function and antioxidants, thereby reducing ROS production. Moreover, we also demonstrated that PGC-1*α* promoted autophagy and inhibited apoptosis in HT-22 cells. Collectively, our findings highlight that the neuroprotective effects of PGC-1*α* on hippocampal neurons are likely to be achieved through multiple targets and mechanisms.

## 5. Conclusions

In summary, our results suggest that PGC-1*α* attenuates the degree of hypoxia-induced hippocampal neuron damage via multiple mechanisms. Thus, mitochondrial function is preserved, and apoptosis is reduced. The regulation of PGC-1*α* expression in hippocampal neurons may be a very promising target for the treatment of cognitive impairment.

## Data Availability

The data used to support the findings of this study are available from the corresponding authors upon request.
